# Workplace violence at emergency departments, Ain Shams University Hospitals, Cairo, Egypt

**DOI:** 10.1186/s12913-022-08867-6

**Published:** 2022-11-28

**Authors:** Altaf O. Assil, Amna A. Salem, Omnya A. Mokhtar, Omnia H. Taha, Amany M. Ramadan, Amal H. R. Mansour, Amal A. E. Awad, Amira A. El-Hossainy, Amir A. Khattab, Alshaymaa M. Salem, Amany E. A. Dalaab, Sonya M. S. Azab

**Affiliations:** 1 Department of Forensic Medicine and Toxicology, Faculty of Medicine, Ain Shams University, Cairo, Egypt; 2 Ministry of Health Hospitals, Cairo, Egypt; 3 Ain Shams University Hospitals, Cairo, Egypt

**Keywords:** Violence, Hospitals, Workplace, Emergency, Physicians, Nurses

## Abstract

**Background:**

The present study aimed to determine the prevalence and forms of workplace violence (WPV) at the emergency departments (EDs) of Ain Shams University Hospitals (ASUH), Cairo and identify risk factors for WPV.

**Methods:**

A cross-sectional study was conducted at the EDs of ASUH comprising attending physicians and nurses using a self-administered structured questionnaire. Interviews were conducted with patients and relatives attending these departments to explore attitudes toward WPV against healthcare workers.

**Results:**

The present study comprised 108 healthcare professionals working in EDs. Verbal violence was the most common type of WPV (86.1%), followed by sexual (48.1%) and physical violence (34.3%). Patient relatives were the most common perpetrator of all types of violence. A lack of facilities was the most common risk factor for violence (82.4%), followed by overcrowding (50.9%) and patient culture (47.2%). On the other hand, approximately 78% of interviewed patients and relatives agreed that the occurrence of violence at EDs was due to several triggering factors, including improper manner of communication by healthcare workers (63.2%), lack of facilities (32.4%), waiting time (22.1%), and unmet expectations (22.1%).

**Conclusion:**

WPV represents a significant issue in EDs with violent behavior against healthcare workers widely accepted by attending patients.

## Background

Workplace violence (WPV) was defined by the WHO as “the intentional use or threatening of using power against a person/group of persons in work-related situations, that either results in or markedly increase the risk for injury, psychological harm, death, mal-development, or deprivation” [1]. One in five healthcare professionals are reportedly exposed to physical violence in the workplace, with a global one year prevalence of 19.33% [2]. WPV impacts on both healthcare professionals and healthcare systems through its effects on the safety, health, and social well-being of healthcare providers [3]. WPV also affects work environment quality and patient care [4].

The reported prevalence of WPV varies according to the definition, type, and measures of WPV used [5] in addition to the location and category of healthcare facilities and staff studied [2]. Emergency departments (EDs) reportedly have the highest prevalence of WPV compared to other healthcare settings due to several factors including work overload, provision of a 24-h service, long waiting times, and interactions with highly distressed patients and relatives [6].

Although WPV is a global issue, significant differences in the causes and specific forms of WPV have been reported between healthcare settings and countries. Further, the prevalence of WPV is reportedly correlated with the prevalence of violence in general society. Accordingly, it is important to consider cultural differences between countries when evaluating the prevalence and causes of WPV [7]. The present study aimed to determine the prevalence and forms of WPV at the EDs of Ain shams University Hospitals (ASUH), Cairo and identify risk factors for WPV by understanding the perspectives of healthcare professionals, patients, and relatives.

## Methods

This was a cross-sectional study conducted at the EDs of ASUH during the period from January to March 2021. The required sample size was calculated using a 95% confidence interval, a power of 80%, response distribution of 50%, and a margin of error of 10%. Accordingly, the estimated required sample size was at least 96 participants.

The study population included all physicians and nurses working in emergency or casualty rooms for a period exceeding one year. Healthcare workers with an employment duration of less than one year were excluded from the present study.

A self-administered structured questionnaire was used to assess exposure of physicians and nurses to workplace violence. The questionnaire included questions regarding the personal characteristics of respondents (age, gender, and occupation), exposure to violence in the workplace in the previous 12 months (verbal, physical, and sexual violence), factors related to incidents of violence, and respondent reactions to exposure to violence. The questionnaire was prepared in Arabic to be understandable to all participants. To explore attitudes toward workplace violence at hospitals among patients and their relatives, interviews were conducted with patients attending EDs and their companions.

SPSS version 19 was used for statistical analysis of collected data. Descriptive statistics (frequency and percentage) and bivariate analysis (Ch-squares test) was used to compare groups. *P*-values less than 0.05 were considered statistically significant.

## Results

The present study included 108 healthcare workers (67 physicians and 41 nurses) from the EDs of ASUH. Table 1 shows the characteristics of the respondents in the present study.

**Table 1:** Characteristics of participating physicians and nurses working at the EDs of ASUH, Cairo, Egypt

Respondent characteristics		Frequency	Percentage
**Gender**	Male	50	46.3
	Female	58	53.7
**Age groups**	< 30 years	70	64.8
	31–40 years	7	6.5
	41–50 years	15	13.9
	No answer	16	14.8
**Job**	Physician	67	62
	Nurse	41	38
**Emergency departments**	Pediatric	25	23.1
	Internal medicine	30	27.8
	Surgery	11	10.2
	Orthopedics	10	9.3
	Obstetrics & gynecology	16	14.8
	Poisoning treatment center	16	14.8
**Total**		**108**	**100**

**Fig. 1:**
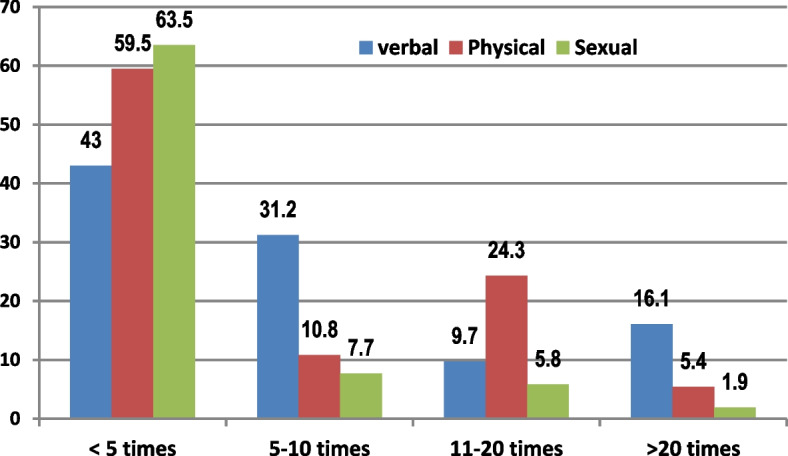
Participants’ exposure to WPV in the last year at the EDs, ASUH, Cairo, Egypt

Table 2 shows the prevalence of different types of WPV against physicians and nurses working at EDs. Verbal violence was the most common type (86.1%), followed by sexual and physical violence (48.1% and 34.3%, respectively). No significant differences in the gender of victims were observed between all types of violence. Most victims of physical or sexual violence reported less than five episodes of exposure to WPV within the previous year, while victims of verbal violence reported a greater frequency of exposure (Fig. 1).

**Table 2:** Prevalence of different types of workplace violence at EDs, ASUH, Cairo, Egypt

Type of violence	**Male**		**Female**		**Total**		**X**^**2**^	***P***
	N	%	N	%	N	%^a^		
**Verbal**	44	47.3	49	52.7	93	86.1	0.278	0.781
**Physical**	16	43.2	21	56.8	37	34.3	0.211	0.688
**Sexual**	21	40.4	31	59.6	52	48.1	1.41	0.253

^a^Proportion of all participants (%, *n* = 108)

Approximately 59% of victims of physical violence (22 participants) reported injuries in the form of simple wounds, while three respondents (8.1%) reported severe injuries (bone fractures in two cases and cerebral concussion in one case).

Table 3 shows the different types of violence reported by participants. Impolite manner of conversation was the most common form of verbal violence (79.6), pushing with hands or kicking was the most common form of physical violence (59.5%), and verbal harassment was the most common form of sexual violence (76.9%).

**Table 3:** Types of workplace violence at the EDs, ASUH, Cairo, Egypt

**Type of workplace violence**	**Forms**	**Frequency**	**%**^a^
**Verbal** **(*n* = 93)**	Impolite manner of conversation	74	79.6
	Shouting/yelling	72	77.4
	Verbal threatening	53	57
	Insults	52	55.9
	Interruption/not listening	40	43
**Physical** **(*n* = 37)**	Pushing with hands/kicking	22	59.5
	Thrown instruments or ER	18	48.6
	Both of the above forms of physical violence	13	35.1
	Assault with weapons	10	27
**Sexual** **(*n* = 52)**	Harassment (verbal)	40	76.9
	Unwanted sexual behavior	16	30.8
	Indecent exposure	3	5.8
	Sexual assault	1	1.9

^a^Proportion of all participants (%)

Table 4 shows the circumstances of incidents of violence at EDs, (ASUH). The patient’s relatives were the most frequent perpetrators in all types of violence, followed by the patients. Male gender was the predominant gender of perpetrators of all types of violence. Night shifts were reported as the more frequent time of incidents of verbal and sexual violence by the greater percentages of victims (45.2% and 42.3%, respectively).

**Table 4:** Circumstances of incidents of workplace violence at EDs, ASUH, Cairo, Egypt

**Circumstances of incidents**		**Verbal**		**Physical**		**Sexual**	
		n	%	n	%	n	%
Perpetrator	Patient companions	74	80	32	86.5	38	73.1
	Patients	30	32	4	10.8	9	17.3
	Others^a^	2	2	1	2.7	5	9.6
Perpetrator gender	Male	55	59.1	19	51.4	25	48.1
	Female	14	15.1	5	13.5	7	13.5
	No difference	24	25.8	13	35.1	20	38.5
Time of exposure to violence	Day shifts	32	34.4	11	29.7	12	23.1
	Night shifts	42	45.2	13	35.1	22	42.3
	No difference	19	20.4	13	35.1	18	34.6
**Total**		**93**	**100**	**37**	**100**	**52**	**100**

^a^Others refers to other coworkers in cases of verbal and physical violence and unknown persons in cases of sexual violence

Regarding incidents of sexual violence, victims reported affection of the perpetrator’s mental state either by disease (19; 36.5%) or intake of drugs or alcohol (14; 26.9%); exposure to sexual violence in hospital settings other than the place of duty (11; 21.1%) as well as multiple perpetrators (≥ two persons) in the same incidents (25; 48%).

Table 5 shows participant reactions to exposure to violence. Most respondents called security to deal with the perpetrators (61.3%) or defended themselves against violent behaviors (47.4%). Only 16.1% of respondents attempted to manage the anger of patients and their relatives.

**Table 5:** Participant reactions to exposure to incidents of violence at the EDs, ASUH, Cairo

**Participant reactions to incidents of violence**	**N**	**%**
Called security	57	61.3
Defended themselves against violent behavior	44	47.4
Attempted to manage patient anger	15	16.1
Escaped	9	9.7
Called police	8	8.6
None	12	12.9

Regarding the effects of exposure to violence, 54.8% of victims (51 respondents) reported a negative impact on workplace performance. Approximately 14% of victims (13 respondents) reported refusal to deliver healthcare to the offending patients.

### Interviews with patients and their relatives

**Fig. 2:**
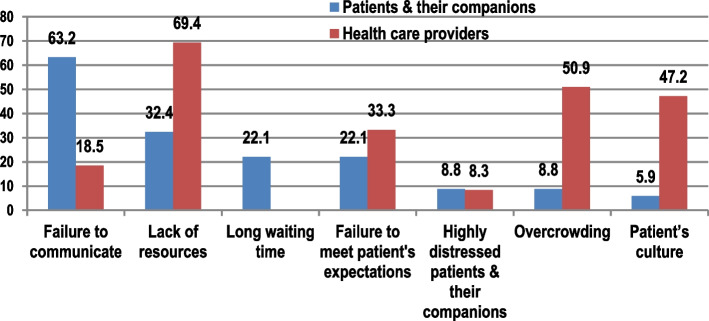
Eliciting factors of WPV at EDs, ASUH, Cairo, Egypt. (The figure shows the reported factors by both of health care workers as well as patients and their companions)

Approximately 78% of interviewed patients and relatives reported observing violence at EDs was a usual and expected result of several triggering factors (Fig. 2). Patients and relatives reported failure of communication by healthcare providers as the most common eliciting factor (63.2%), followed by lack of resources (32.4%), long waiting time (22.1%), and unmet expectations (22.1%). Conversely, healthcare providers reported lack of resources as the most common reason for WPV (69.4%), followed by overcrowding in EDs (50.9%), and patient culture (50.9%).

## Discussion

Egypt is reported to have the highest prevalence of WPV against healthcare workers in Africa (ranging from 59.7% to 86.1%) [8]. The incidence of violence in emergency rooms has been a recurrent problem in Egypt due to lack of security measures at hospitals and the absence of legislation for violence against healthcare professionals. Further, the incidence of WPV reportedly increased during the COVID 19 pandemic [9, 10].

The present study found verbal violence was the most frequent type of violence against physicians and nurses working in the EDs of ASUH, followed by sexual and physical violence. Patient relatives were the most frequent perpetrators of all types of violence. This is consistent with previous studies that found verbal abuse was the most common type of violence in the EDs of Suez Canal University hospital (58.2%) [11] and Tanta University Emergency Hospital (76.5%). Further, patient relatives are reportedly the most common perpetrators of violence in healthcare settings in Egypt [11–14].

The present study found sexual violence was the next most common form of violence at the EDs of ASUH (48.1%). The prevalence of sexual violence in the EDs of other governorates has previously been reported as 30.9% at the Emergency Hospital of Mansoura University [15]; 58.1% at Sohag University Hospital [16]; and 14.12% in Menoufia governorate hospitals [17]. The prevalence of sexual violence to nurses was reported as 70.2% at Tanta University Hospital [18]. This differences may be attributable to work environment and the hospital security systems.

The present study found no significant differences in the prevalence of all types of WPV violence between male and female participants. In contrast, previous studies have reported significant differences in the prevalence of WPV between male and female healthcare workers in Egypt [15, 19] and other countries [20–22].

Of note, 38.5% of victims of sexual violence reported exposure to violence from both genders equally, with 13.5% of respondents reporting females as the predominant perpetrators of sexual violence. Although this is not consistent with local culture and common beliefs, women can initiate violence by using proactively abuse and aggression [23, 24].

The participants of the present study reported a greater frequency of violence during night shifts. Similar findings were reported by Gabr et al. [17] who found nurses in Menoufia governorate hospitals were exposed to a higher frequency of WPV during night shifts compared to day shifts. These findings also corroborate a previous study by D’Ettorre et al*.* (2019) [25] that found an association between frequent night shift work and the occurrence of violence against nurses working at EDs. This finding may be attributable to increased workload affecting the quality of medical care provided by medical staff, thereby provoking WPV by dissatisfied patients [26].

Regarding victims responses to incidences of violence, most respondents called security to deal with the perpetrators or defended themselves against violent behaviors. No respondents filed a formal report against the perpetrator. Similar findings were reported by previous studies that found most of healthcare professionals in Egypt do not take action against perpetrators of violent incidents [10, 11, 14]. Underreporting of WPV is common in healthcare settings worldwide due to several factors including lack of support from supervisors and coworkers, fear of blame or reprisal [27, 28], absence of physical injury, time-consuming procedures for incident reporting [27], and the belief that reporting will not result in positive changes [27, 29]. In addition, medical staff perception of violence as part of their job was found to be a common factor underlying underreporting [6, 27, 30].

Most participants of the present study reported negative effects of exposure to violence on their job performance. This is consistent with previous studies that reported WPV is associated with increased levels of anger, anxiety, depression, and guilt among victims which affects the quality of life of the involved health care professionals [31].

While physicians and nurses participating in the present study reported a lack of resources as the most important risk factor for violence at EDs, whereas patients and their families reported improper manner of communication by healthcare providers as the most important factor relating to WPV. Risk factors for WPV may be related to the assaulted healthcare workers, the perpetrators, and the environment [32]. However, the media and government in Egypt typically blame doctors for all defects and shortages in health care system and reframe them in a negative image which in turn exposes them to aggression and increases the incidence of WPV at hospitals. This was evident during the COVID 19 pandemic when the Egyptian prime minister criticized doctors and claimed they were responsible for the increased deaths among cases of coronavirus. This statement was shocking to doctors and the syndicate condemned the government and warned the statement would increase violence against doctors [33]. Salem et al. (2022) investigated violence against doctors during the COVID 19 pandemic in Egypt and found all participating physicians (100%) believed that the media has a major role in increasing public anger against doctors and exposure of healthcare workers to violence [10].

The lack of resources in Egypt results in stressful conditions in all healthcare institutions. The number of hospital beds in Egypt is 1.4 per 1,000 head of population (while the WHO recommends 5 beds per 1,000 population) [34]. Egypt has one of the Middle East’s lowest ratios of healthcare workers per capita at 0.7 physicians per 1000 head of population and 1.9 nurses and midwives per 1000 head of population [35]. This shortage of human resources is caused by emigration of healthcare professionals from Egypt due to low salaries as well as low quality and stressful working conditions [36].

### Study limitations and strengths

This study was limited by its observational nature and small sample size that limits its generalizability. However, the present study explored the views of healthcare workers and patients toward WPV and risk factors for violence in the same setting.

## Conclusions

Violence within EDs represents a significant problem, particularly as violent behavior against healthcare workers is widely accepted by patients in Egypt. A lack of resources and failure of communication by healthcare professionals were reported as the most common eliciting factors of violence at EDs. The Egyptian government should take action by increasing funding of the healthcare system to overcome the poor work conditions at hospitals. Further, strict legislation should be passed to prevent violence at hospitals. Hospitals should increase security measures and ensure adequate staff coverage to avoid excessive work pressure. Anger management and proper communication with patients and families is mandatory in medical training.

## Data Availability

All data generated or analyzed during the present study are included in this published article.

## Abbreviations

ASUH: Ain Shams University Hospitals
EDs: Emergency departments
WPV: Workplace violence
